# Temporal trends in adolescents’ self-reported psychosomatic health complaints from 1980-2016: A systematic review and meta-analysis

**DOI:** 10.1371/journal.pone.0188374

**Published:** 2017-11-28

**Authors:** Thomas Potrebny, Nora Wiium, Margrethe Moss-Iversen Lundegård

**Affiliations:** 1 Centre for Evidence-Based Practice, Western Norway University of Applied Sciences, Bergen, Hordaland, Norway; 2 Department of Psychosocial Science, Faculty of Psychology, University of Bergen, Bergen, Hordaland, Norway; University of Canberra, AUSTRALIA

## Abstract

**Objective:**

There is increasing concern that mental health may be deteriorating in recent generations of adolescents. It is unclear whether this is the case for self-reported psychosomatic health complaints (PSHC).

**Method:**

We conducted a systematic review and meta-analysis of published primary studies on PSHC in the general adolescent population over time. The primary databases were MEDLINE, Embase and PsycINFO, which were searched from inception to November 2016. Studies were included if they involved an observational design, presented self-reported data from participants aged 10–19 years and included data from at least two time points, five years apart. Inclusion and study quality were assessed by two independent reviewers.

**Results:**

Twenty-one studies were included; 18 reported trends on the prevalence of PSHC in a single country, while three studies reported on multiple countries. In total, over seven million adolescents from 36 countries in Europe, North America, Israel and New Zealand were represented, covering the period 1982–2013. In the descriptive analysis, 10 studies indicated a trend of increasing PSHC, eight showed a stable trend and three showed a decreasing trend at certain points in time. The results from the meta-analysis showed a mean odds ratio (OR) of 1.04 (K = 139, 95% CI 1.01–1.08) for PSHC from 1982 to 2013, thus indicating a minor increase in general. In the subgroup analysis, this minor increase was observed mainly between the 1980s and 2000s, while the trend appeared to be more stable between the 2000s and 2010s. Some differences were also found between multinational subregions. Findings from subgroup analysis, however, only supported a significant increasing trend in Northern Europe.

**Conclusion:**

There may have been a minor increasing trend in adolescent self-rated PSHC between the 1980 and 2000s, but has become more stable since the 2010s, from a multinational perspective. Northern Europe was the only region to show a clearly significant minor increasing trend, without being the region with the highest total prevalence of PSHC at the present time. The discrepant trends regarding PSHC between regions and the reliance on self-reported data may reflect true changes in the occurrence of PSHC in the adolescent population. However, they may also reflect changes in how adolescents perceive and report health complaints.

**Other:**

PROSPERO registration 2016: CRD42016048300.

## Introduction

In recent years, there has been a growing focus on adolescent mental health and well-being in developed countries, with mental health problems now considered to be one of the greatest disease burdens among adolescents, according to the World Health Organization (WHO) [1]. In light of this, there has been increasing concern that adolescent mental health may be deteriorating and that today’s general adolescent population is more at risk from mental health problems than previous generations. The potential increasing risk of mental health problems is problematic as these health problems are universally recognized as having a detrimental impact on adolescents’ well-being, development, academic performance and social capital [2].

Rutter and Smith [3] conducted a comprehensive review on the time trends of psychosocial disorders in young people, showing clear evidence of a substantial increase of psychosocial disorders, including depressive disorders, in developed countries from the 1950s to the 1990s. A systematic review of mental health problems in the general adolescent population from 1983 to 2010 by Bor and colleagues [4] concluded that, while externalizing problems (such as rule-breaking behaviour, drug use and ADHD) appear to be stable, internalizing problems (mental health complaints and symptoms) may be increasing, especially among girls. Further support for the suggested increase in mental health complaints among adolescents between the 1950 and 2010s can be found in several meta-analyses. Twenge and colleagues [5] identified a large generational increase in psychopathological symptoms, including depression, in the course of a meta-analysis among general populations of young people in the US between 1937 and 2007. Xin and colleagues [6] showed that Chinese adolescents’ mental health deteriorated across birth cohorts from 1992 to 2005, reflected in increased scores on the negative indicators of mental health (e.g., mental health problems, anxiety and depression). There is now accumulating evidence that there may have been a real secular increase in adolescent symptom prevalence, although not all the evidence is consistent. This apparent increasing trend in mental health complaints is nuanced by three meta-analyses [7–9], which show a rather stable trend of adolescent mental health complaints and depressive symptoms internationally in non-clinical populations between the 1970s and 2010s.

Mental health complaints have also shown to commonly co-occur and highly correlate with somatic health complaints (reocurring pains and aches) in adolescent population studies [10–13]. Therefore, the combination of mental health complaints and somatic health complaints are often considered to be unidimensional based on empirical- and theoretical grounds [12, 14–17]. At present, there is increasing evidence that somatic health complaints in combination with mental health complaints are important components of mental disorders [18, 19]. Parallel to the development of mental health complaints, there are also clear indications that trends in musculoskeletal pains, or other somatic complaints, among adolescents have been on the increase since the early 1970s to the present decade in Europe [20–22]. Dey and colleagues [13] also found that while levels of mental health complaints showed only minor changes over time, somatic complaints increased monotonically between 1994–2006 in Switzerland.

Indications of increasing mental and somatic health complaints create further concern about deteriorating adolescent mental health problems in more recent birth cohorts, with a fear that there may still be an increasing trend in deteriorating psychological and somatic health at present, compared to the post-war era. Consequently, adolescent mental health problems are now commonly viewed as a pressing global public health issue [2, 19]. Therefore, nowadays, it is of primary importance to identify whether the burden of mental health problems, in combination with somatic complaints, among adolescents is still increasing and, if so, to what extent this affects adolescent health and well-being [4, 23].

The aim of this study is to systematically review the published literature and contribute to the understanding of temporal changes in adolescent self-reported psychosomatic health complaints (PSHC) by investigating differences between birth cohorts in the general adolescent population. Empirically and theoretically, PSHC are regarded as a combination of psychological- (also referred to as mental health complaints) and somatic complaints (reoccurring pains and aches), without a known pathology and without any underlying presumptions about aetiology [12, 19], even though reoccurring complaints are recognized as important indicators of mental disorders [19]. Low levels of PSHC are also considered as good indicators of adolescent psychosocial health and well-being [24].

## Method

This review was structured in accordance with the Preferred Reporting Items for Systematic Reviews and Meta-Analyses (PRISMA) guidelines [25], using the Joanna Briggs Institute (JBI) reviewers’ manual for prevalence and incidence data [26]. A study protocol was published in the International Prospective Register of Systematic Reviews (PROSPERO) in advance of the study (registration #CRD42016048300). The quality assessment for the evidence was evaluated in line with the JBI critical appraisal tool, Checklist for Prevalence Studies [26].

### Inclusion and exclusion criteria

To explore the time trends of self-reported PSHC among adolescent populations, primary studies utilizing a: (1) repeated cross-sectional design/time series (2) within the same geographical area and (3) with similar sampling approaches, which (4) measured a time frame for change over at least two time periods of five years or more, were included in the review. Studies focusing on the general adolescent population and its everyday functioning and well-being meet the inclusion criteria, while studies only measuring psychopathology, suicide incidence and externalizing problems, or solely focusing on “at-risk” adolescent samples were therefore excluded. The adolescent population covered should ideally be nationally representative, either from a national, community or school sample, and recruited using a random sampling methodology. Studies should cover at least parts of the adolescence age range (10–19 years, based on the WHO’s definition of adolescents) and use a broad operationalization of psychosomatic health complaints, i.e., measures of both psychological and somatic complaints and their respective occurrence. In addition, as studies must focus on adolescents’ subjective assessment of their own health using self-reports, measures relying on only parent or teacher reports of adolescent health were excluded in our case. This review was limited to peer-reviewed articles published in English, Norwegian, Swedish or Danish.

### Search strategy

An extensive search for published literature without any historical time or other restrictions was conducted during November 2016. The primary databases searched were MEDLINE, Embase and PsycINFO. The levels of search words used in MEDLINE, in addition to all the relevant subject headings, were as follows: (health complaint* or psychosomatic or psychophysiolog*) OR (subjective or self-reported/ health or complaints) AND (adolescen* or youth or youths or kid or kids or preteen or teen* or child* or young or juvenile) AND (time or trend or trends or secular or temporal). Several complementary searches were performed to insure a sufficiently broad search strategy using an adjusted syntax. These databases were the Web of Science and Google Scholar; for Scandinavian literature, we used SweMed+ and the Norwegian source Helsebiblioteket. A search of the reference list of included studies was also performed. The systematic searches were peer-reviewed by an independent university librarian to ensure the search quality and reduce the risk of selection and detection bias, as recommended by McGowan and colleagues [27], using the PRESS methodology (Appendix 1). The complete search strategy for all the databases is included in Appendix 2.

All identified articles were considered for their relevance to the review, based on the inclusion and exclusion criteria. All the potential articles were read and screened before relevant articles were selected and retrieved. As a first step, relevant articles were considered on the basis of their title and abstract. In a second step, the full text versions of selected papers were examined. All stages of the inclusion process were performed independently by Thomas Potrebny (TP) and Margrethe Moss-Iversen Lundegård (MMIL) using the Rayyan review tool [28]. Agreement was made by discussion between the two authors, based on the inclusion criteria, with regard to final inclusion. Articles meeting the inclusion criteria were then summarized by country, survey, study duration, age groups, measure instrument/scale, and relevant key findings.

### Quality assessment

The assessment of the methodological quality of included primary studies was performed using JBI’s recommended critical appraisal tool, Checklist for Prevalence Studies [26] (Table 1). The appraisal was performed independently by TP and MMIL. Any differences in classification and scoring were discussed until agreement was made and then summarized in the final appraisal. The final quality appraisal can be found in Appendix 3. The assessment was at the study level where possible. If a study contained multiple outcomes only the relevant measure of PSHC was appraised. The quality assessment was used to appraise the overall methodological quality of a primary study and determine the extent to which a study had addressed the possibility of bias in its design, conduct and analysis. If the overall study quality was considered to be low (based on an overall assessment), studies were then excluded from the meta-analysis.

**Table 1:** Critical appraisal checklist.

Was the sample frame appropriate to address the target population?
Were study participants sampled in an appropriate way?
Was the sample size adequate?
Were the study subjects and the setting described in detail?
Was the data analysis conducted with sufficient coverage of the identified sample?
Were valid methods used for the identification of the condition?
Was the condition measured in a standard, reliable way for all participants?
Was there appropriate statistical analysis?
Was the response rate adequate, and if not, was the low response rate managed appropriately?

### Meta-analytic approach

The meta-analysis was conducted using the Comprehensive Meta-Analysis (version 3) software, as developed by Biostat. An OR with a 95% CI was reported as an overall synthesized measure of effect size, representing a ratio of prevalence of PSHC among adolescent populations between two different points in time. Only studies reporting effect sizes that could be converted into an OR were included in the meta-analysis. This included studies that reported either (1) the mean scores of a continuous variable with standard deviation/standard error or (2) the outcome as a binary/dichotomized variable with rates or counts, combined with sample size. While studies that provide correlational data can also be included in a meta-analysis, this option was not applicable for this review. As long as the underlying continuous or binary measurements are considered to follow a similar logistic distribution (for example, as with depression), it is possible to re-express the outcomes as a common effect; in this case, OR [29, 30]. Even if these assumptions do not hold exactly, Borenstein and colleagues [30] advise against simply omitting certain studies, as this would involve loss of information and possibly a *systematic loss* of information, which could result in a biased sample of studies. Upon making these decisions, performing a sensitivity analysis is recommended [30]. To facilitate conversion to a common metric, each study was first computed to an individual effect size and variance of its native index (log OR for binary data and d for continuous data). Second, they were all converted to a common index, which was a log OR. Finally, using the exponential function, transformation to the final OR was performed and presented [30].

As considerable heterogeneity was expected between studies, pooled mean effect size was calculated using the random effects model. Random effects models are recommended when accumulating data from a series of studies where the effect size is assumed to vary from one study to the next, and where it is unlikely that studies are functionally equivalent [30]. The Q^within^ statistic was used to assess the heterogeneity of studies. A significant Q^within^ a value rejects the null hypothesis of homogeneity. An I^2^ statistic was computed as an indicator of heterogeneity in percentages. Increasing values show increasing heterogeneity, with values of 0% indicating no heterogeneity, 50% indicating moderate heterogeneity, and 75% indicating high heterogeneity [31].

**Fig 1:**
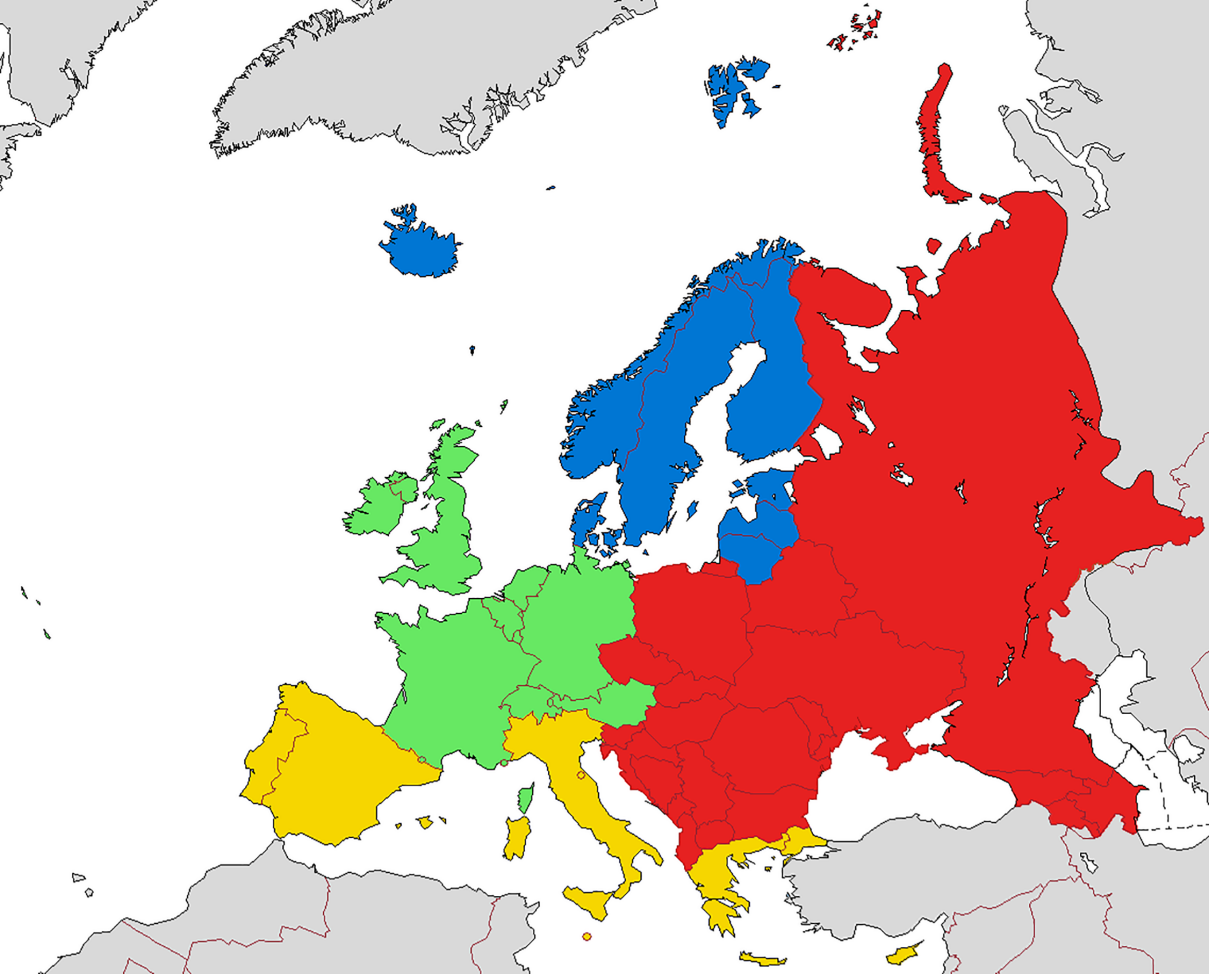
European subregions defined by EuroVoc. Blue—Northern Europe, green—Western Europe, red—Eastern Europe, yellow—Southern Europe, grey—territories not considered part of Europe. By Samotny Wędrowiec, 2014, via Wikimedia Commons. Used under Creative Commons Attribution-ShareAlike 3.0.

Subgroups of gender and complaint type (psychological and somatic) were grouped together where applicable for the main analysis. Several additional subgroup analyses were planned by gender, age group, complaint type, country/region and historical time point, where possible. As we hypothesized that differences in macro-level determinants of health between regions, such as social, political and cultural influences, could influence PSHC and contribute to different trends, the study samples were divided into global subregions. Samples from the European continent were pragmatically divided into groups based on EuroVoc, the official thesaurus of Europe [32]. Greenland was added to Northern Europe due to its close political and historical history with Europe. This resulted in a subset of Northern, Eastern, Southern and Western Europe. Definitions of European subregions are illustrated in Fig 1. Samples from North America were also grouped, while the remaining samples from New Zealand and Israel were grouped as “other subregions”.

Studies that measured PSHC at more than two time points were grouped in order to compare trends during specific time periods. To make all the studies comparable, and to include all available information about the time trends, the prevalence of PSHC at the first measured time point was compared to the second, then the second to the third, and so on. This gave an OR that estimated the *relative change* in trends in the prevalence of PSHC within the data for the main meta-analysis.

## Results

### Descriptive findings

**Fig 2:**
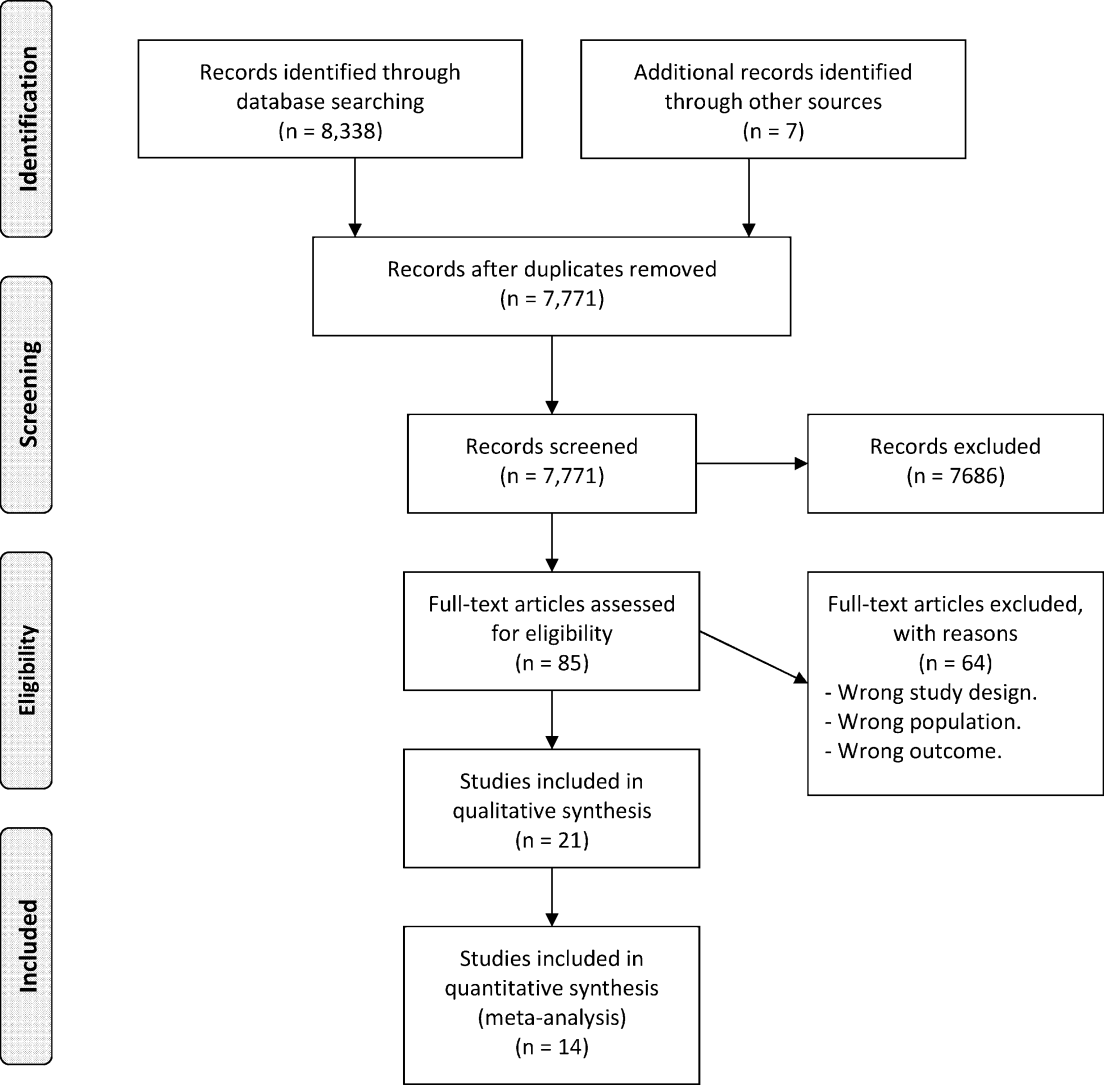
Flow diagram for the study selection process.

The formal systematic search of the literature yielded 8.338 potentially relevant articles, while complementary searches yielded only seven articles. After duplicates were removed, 7.771 remained for screening. Upon reviewing the title and abstract, 85 articles were considered to be potentially relevant and their full text was assessed for eligibility. Sixty-four out of the 85 articles were excluded due to (exclusively or in combination) wrong study design, outcome or population. Thus, 21 articles met the inclusion criteria and were included in the final qualitative synthesis. For the meta-analysis, 14 studies provided the necessary outcome needed to be included in the analysis (Fig 2). While all samples within the studies were independent of each other, there was some overlap in the data set between studies within the “HBSC” survey and the “Young in Värmland” survey. Thus, in the meta-analysis, only one measure for each independent sample was used. The sample with the most consistent outcome was included, while the remaining studies were excluded.

Out of the 21 included studies, 18 studies reported on trends in the prevalence of PSHC in the general adolescent population over a period of at least five years in *single countries* [13, 19, 33–48], while three reported on *multiple countries* [8, 24, 49]. Out of the three studies that examined trends in multiple countries, one reported data from five Nordic countries [49], while two reported comprehensive international summaries of the HBSC survey that included data from 35 countries across Europe, North America and Israel [8, 24]. Overall, 36 countries, mainly in Europe, as well as the USA, Canada, Israel and New Zealand, covering the time period 1982–2013 and representing a sample of over seven million children and adolescents and their experiences of health, were included in the current review (Table 2).

**Table 2:** Summary of studies included in the review.

Lead author	Country (Survey)	Study duration (years)	Age group (years)	N	Outcome measure/scale	Mean score^a^/ Categorical	Trend type^b^	Included in Meta-analysis	Relevant key findings & sampling procedure
Achenbach (2002) [33].	USA (Other)	1989, 1999	11–18	2.737	YSR (internalizing symptoms scale).	M	S	No	• No trend of significant change was observed in PSC, and no differences between genders or age, over time. • Mean levels of complaints were reported to be high. • Indicates that complaints may be higher than in other comparative cultures. • - Stratified random sampling procedure (based on 48 states).
Berntsson (2001) [49] & Berntsson (2014) [34].	Nordic countries (NordChild)	1984, 1996, 2001	2–17	21.997	Generic PSC scale.	> 1 complaint every/other week.	I	Yes	• An increasing trend of PSC over time. • Higher prevalence among girls than boys. • Children (age 2–6) have lower PSC than adolescents (Age 7–12 and 13–17). • No significant difference between early- and late adolescent age groups. • Stratified random sampling procedure (based on age and gender).
Braverman (2016) [35].	Norway (HBSC)	1994, 1998, 2002, 2006, 2010	15	7.761	HBSC-SCL.	M	I	Yes	• An increase trend of PSC over time. • Higher prevalence among girls than boys. • Cluster random sampling procedure (based on school or class).
Dey (2015) [13].	Switzerland (HBSC)	1994, 1998, 2002, 2006	11–15	33.625	HBSC-SCL.	M	S	Yes	• A trend of fewer 'psychological' complaints, while there was an increasing trend of 'somatic' complaints. • Different trajectories for the psychological and somatic health complaints. • Higher prevalence of complaints among girls and older adolescents. • Cluster random sampling procedure (based on school or class).
Due (2003) [36].	Denmark (HBSC)	1988, 1991, 1994, 1998	11–15	12.782	HBSC-SCL.	> 4 complaints/week	S	No	• No consistent change in trends of PHC. • Cluster random sampling procedure (based on school or class).
Duinhof (2014) [37].	Netherlands (HBSC/DSSNU)	2003, 2005, 2007, 2009, 2013	11–16	29.352	SDQ (emotional symptoms scale).	M	S	Yes	• No change in the trend of PHC was observed. • Boys have a significant but negligible (according to the authors) different trend of PHC over time than girls. • Higher prevalence among girls than boys, also negligible. • Cluster random sampling procedure (based on school or class).
Fink (2015) [38].	England (Other)	2009, 2014	11–13	3.366	SDQ (emotional symptoms scale)	M	I	Yes	• A trend of increasing PHC over time. • Different trend between girls and boys. • Higher prevalence among girls than boys. • Non-probability sampling procedure (based on 200 schools).
Fleming (2014) [39].	New Zealand (NAHS)	2007, 2012	13–18	17.607	SDQ (emotional symptoms scale)	> 7 subscale score	I	Yes	• A trend of increasing PHC over time. • Higher prevalence among girls than boys. • Cluster random sampling procedure (based on schools).
Hagquist (2009) [40], Norell-Clarke (2016) [45] & van Geelen (2016) [19].	Sweden (Young in Värmland)	1988, 1991, 1995, 1998, 2002, 2005, 2008, 2011	15–16	20.115	PSP	M and > 90^th^ percentile	I	Yes	• A trend of increasing PHC over time. • Different trend between girls and boys. • Higher prevalence among girls than boys. • Total population sample procedure (Based on all 15–16 year olds in the county).
Henriksen (2012) [41].	Denmark (Other)	1996, 2010	6–16	949	YSR (internalizing symptoms scale)	M	I	Yes	• A trend of increasing PHC over time. • Boys have a larger increase of PHC over time then girls. • Higher prevalence among girls than boys. • - Non-probability sampling procedure (based on two schools).
Levin (2009) [42].	Scotland (HBSC)	1994, 1998, 2002, 2006	11–15	19.393	HBSC-SCL	> 2 complaints/week	D	No	• Decreasing trend of PHC over time. • Increasing trend of mental well-being. • Indications of increasing socioeconomic inequalities in health and well-being. • Higher prevalence of PHC among girls than boys. • Cluster random sampling procedure (based on school or class).
Levin (2015) [43].	Scotland (HBSC)	1998, 2002, 2006, 2010	11–15	18.470	HBSC-SCL	Never-daily	D	No	• A Likely decreasing trend of PHC over time. • Cluster random sampling procedure (based on school or class).
Maughan (2012) [44].	UK (UK national studies by ONS)	1999, 2004	5–15	18.415	SDQ (emotional symptoms scale)	M	D	Yes	• Decreasing trend of PHC over time. • No difference in prevalence between genders. • Stratified random sampling procedure (based on population registries).
Ottová-Jordan (2015a) [8].	International (HBSC)	2002, 2006, 2010	11–15	510.876	HBSC-SCL	M	S	No	• Fairly stable international trend of PHC. • Higher prevalence among girls than boys, across all countries and age groups. • Higher prevalence among older adolescents. • Cluster random sampling procedure (based on school or class).
Ottová-Jordan (2015b) [24].	International (HBSC)	1994, 1998, 2002, 2006, 2010	15	237.136	HBSC-SCL	> 2 complaints/week	S	Yes	• Rather stable international trend of PHC across countries, but great variation in prevalence rates between countries and survey years. • Cluster random sampling procedure (based on school or class).
Sourander (2012) [46].	Finland (Other)	1998, 2008	13–17	3.027	SDQ (emotional symptoms scale)	M and >90^th^ percentile	S	Yes	• No change in the trend of PHC was observed. • Higher prevalence among girls than boys. • Total population sample procedure (based on all 13–14 year olds in two cities).
Tick (2008) [47].	Netherlands (Other)	1993, 2003	11–18	1.905	YSR (internalizing symptoms scale)	M and >84^th^ percentile	S	Yes	• Different trends for boys and girls in PHC. Girls showed an increase over time (mainly for somatic complaints), while boys had a decrease (the decrease was mainly for somatic complaints although boys showed a parallel significant increase of psychological complaints). • Higher prevalence among girls. • Simple random sampling procedure (based on population registries).
Twenge (2015) [48].	USA (Other)	1982–2013 • (yearly)	14–18	6.900.000	Generic PSC scale (similar to CES-D)	M	I	Yes	• A trend of increasing PHC over time. • Different trend between girls and boys. • Different trajectories for the psychological and somatic health complaints (higher increase of somatic complaints). • Multi-stage random sampling procedure (based on schools).

^a^ Mean score = M

^b^ I = increasing-, D = decreasing-, S = stable trend

### Risk of bias assessment

Overall, the risk of bias was considered to be low for the included studies, with the exception of Henriksen’s study [41], which had a lower, albeit adequate, level of methodological quality. All studies were observational and had a repeated cross-sectional design, which is common in the field. Most studies utilized a random sampling method, had a large sample size (of at least 900 adolescents) and were nationally representative. In general, the external validity was therefore considered to be high, but there was a skewness of studies focusing exclusively on age 15, thereby reducing the generalizability to other adolescent age groups. The outcomes, which were typically assessed by questionnaires completed by the adolescents, consisted of a measure of PSHC that reflected both psychological and somatic health complaints and was assessed using the validated scales: Youth Self-Report (internalizing symptoms scale), HBSC Symptom Checklist, Strength and Difficulties Questionnaire (emotional symptoms scale), Psychosomatic Problems Scale and other similar non-validated “generic” PSHC scales. The full quality appraisal with comments can be found in Appendix 3.

### Meta-analysis

#### Overall results, including by decade

**Fig 3:**
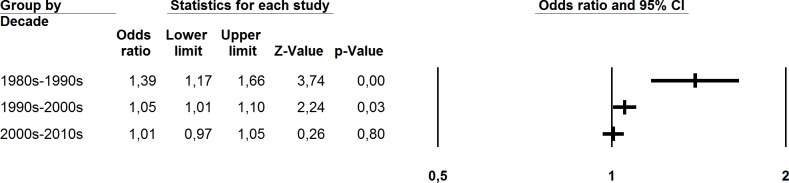
Forest plot for meta-analysed studies on adolescents’ PSHC, grouped by decade.

Of the 21 included studies, 10 indicated a trend of increasing PSHC, eight showed a stable trend and three showed a decreasing trend over time at certain points in time between 1982 and 2013. In the meta-analysis, a synthesis of 139 independent samples with measurements at two time points between 1982 and 2013 provided an overall OR estimate of 1.04 (95% CI 1.01–1.08). High levels of heterogeneity were established for the effect sizes included in this overall estimate (Q^within^ 1156.15, p<0.01, I^2^ = 88.06) (Table 3). Due to the hypothesized difference in effect between the historical time points reported in previous studies, a subgroup analysis was performed for the different decades in an attempt to explain some of the high heterogeneity in the main analysis. The analysis showed that differences in PSHC trends between decades were significant (Q^between^ 13.80, df = 2, p<0.00), when comparing studies carried out between the 1980s and 1990s (K = 8, OR 1.39, 95% CI 1.17–1.66) to studies carried out between the 1990s and 2000s (K = 57, OR 1.05, 95% CI 1.01–1.10) and studies carried out between the 2000s and 2010s (K = 74, 1.01, 95% CI 0.97–1.05) (Fig 3 and Table 3). The findings indicate possibly different trends in PSHC for the different decades. The subset of studies carried out between the 1980s and 1990s had a significantly higher OR than the two following decades. From the 1980s until the 2000s, there appears to have been an overall increasing trend at a multinational level, while the trend between the 2000s and 2010s appears to be more stable. Achenbach and colleagues [33], whose findings were not included in the meta-analysis due to not presenting comparable scale scores, also reported a small, but non-significant, increase among US adolescents from 1989 to 1999.

**Table 3:** Meta-analytic findings for studies on adolescents’ PSHC (Random effects model).

	K	Mean OR	95% CI	Q	I^2^
Overall estimate	139	1.04^b^	1.01–1.08	1156.15^a^	88.06
By decade					
1980s-1990s	8	1.39^a^	1.17–1.66	112.78^a^	93.79
1990s-2000s	57	1.05^b^	1.01–1.10	372.91^a^	84.98
2000s-2010s	74	1.01	0.97–1.05	561.88^a^	87.01
Total between				13.80^a^	

^a^ P<0.001.

^b^P<0.05.

### Multinational subregions

**Fig 4:**
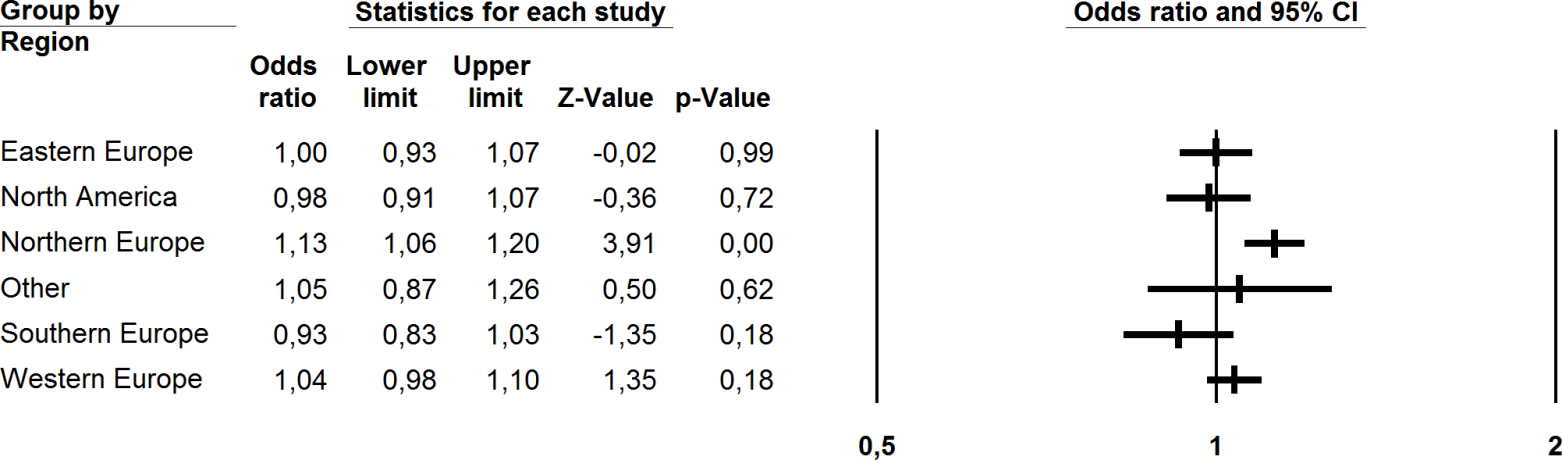
Forest plot for meta-analysed studies on adolescents’ PSHC, grouped by multinational subregion.

Based on hypothesized differences between countries and subregions, and on the noticeable differences in trends of adolescent PSHC between countries and subregions in findings from descriptive analyses, a subgroup analysis was performed on studies from different multinational subregions. From the analysis, differences in PSHC across subregions were significant (Q^between^ 13.83, df = 5, p<0.02) when comparing studies from Eastern Europe (K = 26, OR 1.00, 95% CI 0.93–1.07), North America (K = 13, OR 0.98, 95% CI 0.91–1.07), Northern Europe (K = 46, OR 1.13, 95% CI 1.06–1.20), Southern Europe (K = 10, OR 0.93, 95% CI 0.83–1.03), Western Europe (K = 39, OR 1.04, 95% CI 0.98–1.10) and other regions (K = 5, OR 1.05, 95% CI 0.87–1.26). In general, only small differences existed between subregions. However, the Northern European region showed a clear, albeit minor, increasing trend with regard to PSHC among adolescents (Table 4 and Fig 4).

**Table 4:** Meta-analytic findings for studies on adolescents’ PSHC, by multi-national sub-region (Random effects model).

	K	Mean OR	95% CI	Q	I^2^
By sub-region					
Eastern Europe	26	1.00	0.93–1.07	160.67^a^	84.44
North America	13	0.98	0.91–1.07	122.74^a^	90.22
Northern Europe	46	1.13^a^	1.06–1.20	309.47^a^	85.46
Other	5	1.05	0.87–1.26	38.97^a^	89.74
Southern Europe	10	0.93	0.83–1.03	49.41^a^	81.79
Western Europe	39	1.04	0.98–1.10	395.98^a^	90.40
Total between				13.83^a^	

^a^ P<0.001.

### Gender and complaint type

Nearly all studies reported girls having a higher prevalence of PSHC than boys. While the majority of studies show that the higher prevalence of PSHC among girls was stable over time, there are also several indications of a different trend development between girls and boys. Some studies indicate that increasing PSHC are found in girls, but not to the same extent in boys, over time [37, 38, 40, 47, 48], while Henriksen [41] found the opposite, that is, that boys have an increasing trend of PSHC compared to girls. However, based on a subgroup analysis of these study effects, there was no clear evidence of a significant difference in trends for boys and girls, which is also in line with most of the studies not included in this analysis. That said, we cannot exclude the possibility of different gender trends due to the small number of comparisons available for this analysis (K = 26).

In preliminary analysis, two studies also showed that there may be a different trend over time for psychological versus somatic health complaints among adolescents [13, 48], while one study reported no differences between the two dimensions of complaints [35]. A subgroup analysis showed no clear difference in trends of psychological and somatic health complaints (K = 26). Although previous research has suggested a two-factor solution [12, 13], PSHC can also be conceived as measuring the unidimensional latent traits of psychological and somatic complaints [14].

### Sensitivity analysis, publication bias assessment and other notes

Performing a sensitivity analysis is recommended in order to test the robustness of the findings, when combining continuous and categorical measures in a meta-analysis [30]. Important assumptions to test are the assumption that the effect from continuous measures does not differ from the results of the categorical measures, and the assumption that the result of the meta-analysis will not differ with or without the categorical measures. The meta-analysis consisted of 139 samples with categorical measures and 20 samples with continuous measures; there was no significant difference between these samples (Q^between^ 0.24, df = 1, p = 0.62). When the samples with continuous measures were removed, the main meta-analysis still showed similar results (K = 119, OR 1.04, 95% CI 1.00–1.08, p<0.04). One study had a higher risk of bias, but the results of the main analysis did not change, even when bias was removed from the study [41]. Comparing the results of the different measurements used in the included studies showed no significant differences between measures, except for the “generic” psychosomatic health complaint scales showing a larger effect size compared with other measurements. This applies specifically to only two studies, Berntsson and Köhler [49] and Twenge [48], both examining trends from the 1980s and onwards, a period in which adolescents’ mental health may have been deteriorating, as shown also by other researchers [3]. The probable increase of PSHC during this period may explain some of the dissimilarities between the “generic” measures and other measures of PSHC.

Four indicators of publication bias were examined: Rosenthal’s fail-safe N, funnel plot, Duval and Tweedie’s trim and fill procedure, and Egger’s regression intercept [30]. Rosenthal’s fail-safe N indicated that 1,815 missing studies were needed to make the overall measure non-significant. Following the recommendations for interpretations by Sterne and colleagues [50], a funnel plot indicated relative symmetry, but with additional horizontal scatter, which was likely attributed to the high heterogeneity resulting from the differences between studies (in terms of different country and time point). When interpreting the funnel plot, Egger’s regression test showed that the intercept was not significantly different from zero (B0 = -0.15, 95% CI -1.29–1.00, p = 0.80), while Duval and Tweedie’s trim and fill method indicated that there were no missing studies to the left or right of the mean. This suggests that the impact of potential publication bias is trivial [30].

Two final comments are worth considering. In one study [49], the raw OR scores of all ages between two and 17 years of age, were reported in the primary study. For the meta-analysis, it would have been preferable to only report on the adolescent age group (10–19 years) in the different countries. However, the OR was not significantly different for the two to 17 years age group and the 13 to 17 years age group. Since children and younger adolescents have been shown to have lower amounts of health complaints than older adolescents [4], the mean OR in the meta-analysis may therefore have had a lower mean OR than the 10 to 17 years age group, as indicated by the descriptive statistics in this primary study. Finally, it should be noted that we were not able to treat adolescent age and historical time points as continuous measures as this data was not available in the included studies. As such, further analysis could not be performed.

## Discussion

### Increasing trend of psychosomatic health complaints among adolescents

This review of secular trends among adolescents’ self-reported PSHC from 1982 to 2013 examined 21 epidemiological studies with an overall low risk of bias. Out of the 21 included studies, 18 studies reported trends in the prevalence of PSHC in the general adolescent population over a period of at least five years in a single country, while three studies reported on multiple countries. Over seven million adolescents from 36 countries in Europe, North America, Israel and New Zealand were represented. In the descriptive analysis, 10 studies indicated a trend of increasing PSHC, eight showed a stable trend and three showed a decreasing trend at certain points in time between 1982 and 2013. Overall, results from the meta-analysis indicated that trends involving PSHC in adolescence vary between country, multinational subregion and historical points in time from which studies were carried out.

The results from the meta-analysis showed a mean OR of 1.04 for PSHC between 1982 and 2013, thereby indicating a minor increasing trend in general. In subgroup analysis, this minor increasing trend was observed mainly from the 1980s to the 2000s, while the trend appeared to be more stable during the 2000s to the 2010s. Some differences were also found between multinational subregions, where some regions showed an increasing trend regarding PSHC (e.g., Western Europe, Northern Europe and “other regions”, that is, New Zealand and Israel), while others showed a more stable or decreasing trend (e.g., Eastern Europe, Southern Europe and North America). However, findings from the subgroup analysis only support a significant increasing trend in Northern Europe.

In the same time frame as this review, there have also been some notable indications of congruent trends among general adolescent populations in terms of increasing symptoms of mental health disorders, musculoskeletal pains and the increasing use of health services, for the diagnosis and treatment of both psychosomatic health complaints and mental health disorders, in high-income countries [23, 48, 51], which broadly supports the findings from this review. Not all findings are as consistent however. Several researchers have noted the contrasting evidence between increasing adolescent mental health issues over the last decades, compared with the improvement in favourable prerequisites for adolescents’ health and well-being [23, 52]. This has led to an ongoing debate in terms of both the reliability of the present evidence, but also the presumed severity and impact on adolescent physical health, impairment and school absenteeism. At present, it is unclear whether the change in self-reported PSHC represents an actual change in adolescents’ everyday functioning, physical health and well-being [23, 53]. It has been argued that the two pathways are not mutually exclusive and that the increasing *risk* of mental health issues may not necessarily exacerbate physical health and well-being [3, 23]. These observations are also in line with the theoretical notion that the concept of illness can be differential to the concept of sickness and disease, while there is an inevitable overlap between these distinct dimensions of health [54]. Regardless, the implication in the case of adolescent mental health is that the increasing prevalence of PSHC or mental health problems may not actually lead to any significant increase in adolescent impairment. One of the recent studies included in this systematic review examined the trend of adolescent PSHC in combination with functional impairment between 1988 and 2011 in Värmland, Sweden [19]. Functional impairment was operationalized as a composite score of the high degree of school absenteeism, difficulty on most or all school courses, and never or rarely experiencing any social activity with family and peers. The study found that the PSHC not only increased in this time period along with functional impairment, but this increase also contributed to a significantly amplified risk of functional impairment over time. The authors concluded by stating the notion of a time trend of deteriorating adolescent mental health and that the long-term findings in this field “constitute a real and pressing health priority” (p. 55).

A longitudinal study from Norway also indicates that high levels of adolescent PSHC do indeed increase the risk of functional impairment in the transition from adolescence to young adulthood, in the form of sickness absence and the use of medical/welfare benefits [55], which could in turn increase the likelihood of young adults being marginalized from the labour market all together [56]. The failure to complete secondary school education, which is also regarded as a major public health challenge in high-income countries, is also associated with adolescent PSHC and everyday impairment [55, 57]. It is therefore likely that strategies targeting PSHC in adolescence also have the potential to reduce the future receipt of medical/welfare benefits in young adulthood, as well as secondary school incompletion rates [55, 58].

In light of this, it is quite understandable that there is, in part, a worldwide concern that adolescent mental health could be deteriorating and that today’s young people may be more prone to mental health problems than previous generations [2, 19]. There is a need for studies on impairment in adolescents’ everyday functioning or other measures of well-being to assess whether adolescent mental health is further deteriorating.

### The curious case of Northern Europe

Interestingly, Northern Europe showed a different trend than other multinational subregions. Indeed, it was the only region that showed a clearly significant minor increasing trend in adolescent PSHC between 1982 and 2013 (K = 46, OR 1.13, 95% CI 1.06–1.20). This increasing trend is also apparently similar in all nine Northern European countries included in this review across the Nordic countries, including Greenland and the Baltic states. Demographically, countries in Northern Europe are considered to have a very high human development index (HDI) [59], while the Nordic countries are mostly considered to be leaders in promoting health through public policy [60]. It has previously been argued that, while the Nordic countries have excellent prerequisites for adolescent health and well-being, PSHC still appear to be increasing, which is something of a paradox [52]. While the same paradox may seem to be apparent in this review, one nuancing factor is also observed in our data: the increasing relative trend of PSHC in Northern Europe does not necessarily indicate that the prevalence of complaints is highest overall. For instance, there are strong indications of different time trends regarding PSHC when comparing the USA and Norway [8]. This different trend regarding PSHC does not necessarily account for their current prevalence. In 1994, the rate of multiple recurrent PSHC in Norway was 21.8%, while there was a monotonic increase to 32.5% in 2010. In contrast, the USA showed a rather sharp declining trend of PSHC from 45.7% in 1994 to 36.9% in 2010 [24]. This illustrates that, even though these countries have contrasting trends that differ completely, the prevalence of PSHC appears to be higher in the USA compared to Norway. This may suggest that, even though the trend of increasing PSHC in Northern Europe is of considerable public health concern, accounting for differences in multinational prevalence may nuance the findings of this review somewhat.

### Social determinants of psychosomatic health complaints

The nature of the studies reviewed does not allow for any causal inference. It is agreed that a myriad of factors will have influenced adolescents’ mental health between 1982 and 2013, while the very nature of explaining the temporal trends is a complex task [3, 23]. However, there are some suggestions with regard to possible determinants of PSHC that are worth noting. In general, when it comes to social determinants of psychosomatic health across time and country, it is difficult to identify any particular meaningful change, as there are many small changes in both proximal factors, such as individual health behaviours and distal factors (changes in country-level factors), such as gross domestic product (GDP) [8]. What is suggested, however, is that variations in PSHC can mostly be explained by individual factors, which have been comparatively stable over time [8, 61]. Thus, rather than changing factors, we should talk about stable factors. The main determinants showing a clear association with PSHC in the data from 2002 to 2010 are: being a girl, having been bullied, smoking, and experiencing school-related pressure [8]. Negative effects relating to poor social networks, peer socialization and high (electronic) media use have been shown to be associated with increased PSHC [34, 61–63]. Increasing screen time use in adolescence may also have changed the way that adolescents relate to physical activity and socialization among peers [61, 64]. The apparent negative effect of high electronic media use may also be more pronounced among adolescent girls, suggesting gender-specific intervention programmes aimed at this population may be appropriate [61].

Nearly all studies in this systematic review reported a higher prevalence of PSHC among adolescent girls than boys. This gender effect appears to be mostly stable over time, although there are several indications of divergent trends in line with the findings of Bor and colleagues [4], who concluded that the burden of internalizing symptoms is increasing among adolescent girls, possibly more than for boys; this may warrant further investigation. In any case, it has been well established that there are gender differences in rates of health complaints among adolescent girls and boys. One reason highlighted for the difference in subjective health between genders is that girls are somewhat predisposed to a higher extent than boys [4]. It also may be, in part, due to increasing school-related pressure, earlier sexualization, earlier onset of puberty and other societal changes, such as media and consumer culture, which is presumed to negatively affect adolescent girls to a higher extent than boys [3, 4, 8, 49, 54].

While PSHC are often considered to negatively affect girls more than boys, one could conversely expect that adolescent boys tend to have more externalizing problems (such as conduct disorders, ADHD and illicit drug use). Bor and colleagues [4] indicated that externalizing problems appear to be relatively stable. Although externalizing problems is outside of the scope of this systematic review, several of the included studies also reported adolescent self-reported externalizing problems. Two of these studies showed no increase in general externalizing problems [33, 41], while one showed a small increase in externalizing problems among boys [47].

### Macro-level determinants

As stated earlier, the trends of prevalence of PSHC vary between countries, multinational subregions and historical time points. One can only theorize about the direct influence of macro-level determinants on adolescent PSHC in recent history. Although proximal factors seem to have a larger effect than distal factors (such as GDP, country-level income distribution (Gini index), HDI and changing values, which, to a certain extent represent the modernization of society), there are still indications of a macro-level impact on young people’s health, despite the presence of discrepant results [8, 61, 65]. Distal macro-factors, such as the change in the orientation of values from the collective to the individual, changing family structures and the overall modernization of society, have evolved, which could also have contributed to changes in adolescent PSHC trends [3, 64, 66]. Bremberg [67, 68], however, argued that part of this modernization, such as changes in family structure and values, cannot explain the variation between European countries. Bremberg instead hypothesized that increased competition in the labour market increasingly demands higher qualifications by means of extended education. By extension, young people who fall out of the school system at a young age and who are not established in the labour market may have a heightened risk of health complaints.

Other authors have highlighted the importance of healthcare systems and health policy as determinants of adolescent health, as there has been substantial development in child and adolescent mental health services- and policy during the last decades [69]. Patel and colleagues however, critically argue that healthcare system responses to emerging youth mental health issues have been inadequate despite the positive development. Supportive evidence for this argument is found in a review of international adolescent mental health policy that showed that only 7% of WHO member countries (14 of 191) had clearly articulated child and adolescent mental health policy before the year 2004 [70]. Shatkin and Belfer therefore concluded that few healthcare systems and health policies were designed to fully support adolescent mental health issues. However, most of the countries that did have identifiable policies recognising the developmental and mental health needs of youths were in Europe, in line with other studies [60, 71]. The European healthcare systems, for example, have seen quite a dramatic increase of skilled healthcare professionals over the last decades as a result of its health policies, while North American and New Zealand’s health policies aimed at young people’s mental health have also been given greater priority since the 1980s [70, 71]. The situation within different counties however, remains very heterogeneous with regard to the organization of health services.

The question remains whether the advancement in health policy in developed countries combined with the increase of professional health workers has led to greater utilization of mental health services among the youths in these countries. There are several studies indicating that the majority of young people with higher levels of health complaints or with mental health problems, tend not to seek help from available health services—in Europe and North America [72–77]. Patel and colleagues [69] argue that in the context of developed countries, services tailored to young people remain scarce and are not sufficiently “youth friendly”, that is, encouraging help-seeking behaviour. Furthermore, young people’s health problems are often diagnostically confusing, and often need multidisciplinary and intersectoral responses. For these reasons, the authors suggest that a substantial gap exists between efficacy and effectiveness in mental health care for young people. It may therefore be important to advocate healthcare systems that act to continually improve multidisciplinary and intersectoral action through health promoting policy, to be able to counteract potential trends of increasing adolescent health complaints and mental health problems and to support positive youth development.

Whatever the case may be, macro-level factors’ influence on PSHC are likely to function indirectly through mediating factors, such as social and public policy [8]. Therefore, an analysis of macro-factors may enrich the understanding of adolescent health trends, which is of critical importance when developing public health policy, especially since Patton and colleagues [2] suggest that adolescent health has not explicitly been prioritized in the effort to secure health equity.

### Limitations

There are several limitations in this systematic review that should be noted. One limitation is the reliance of self-reported studies from adolescent themselves, which rely on symptoms and behaviour ratings, rather than more objective measures of health. This, however, has provided the current authors with the opportunity to look at patterns of trends over an extended period of time, which have otherwise been unavailable for research. Furthermore, the number of studies eligible for inclusion was small, while studies with somewhat different outcome measures were included. Only including studies using the same measure of health complaints limits the overall narrative understanding. This limitation does, however, highlight the need for studies that utilize consistent methods and cover both the psychological and somatic aspect of subjective health across multiple cohorts. Several research studies have noted that self-reported health measures are valuable to public health research [4, 23]. Self-reported health complaints have also been shown to reliably predict future mental illness [19]. The discrepant trends in PSHC between regions and the reliance on self-reported data may reflect true changes in the occurrence of PSHC in society; however, they may also reflect changes in subjectivity over time and how adolescents perceive and report health complaints. Changes in the way of reporting *have* likely been an influencing factor over time, although it is regarded as unlikely that this is the main explanatory factor [4, 68].

Investigating psychometric properties of the PSHC measurements showed some indications of differential item functioning (DIF) for certain PSHC items. DIF was, for example, found over time in a Swiss sample, for the item “sleeping difficulties” [13]. However, no discernible DIF was found for a similar item over time in a Swedish sample [15]. Hagquist [15] states that the PSHC measurement adequately meets measurement criteria and proper categorisation of the items included, and does not recommend removing any items in future trend analysis. In a cross-national analysis however, the item “sleeping difficulties” showed a sizeable DIF when compared across countries within the HBSC-study. Thus, the authors recommend discarding this item in future research comparing PSHC cross-nationally [14]. There were also some indications of DIF in the item, “dizziness”, which may be attributed to linguistic differences between socio-demographic subgroups in Switzerland [13]. In sum, these analyses on psychometric properties indicate that even though certain items may have introduced bias, especially in comparisons of complex cross-national data, the impact of this potential bias is currently not known [13]. Future research on DIF over time in population studies on adolescent health is therefore recommended.

## Conclusion

This review observed a minor increasing trend regarding adolescent self-rated PSHC between the 1980 and 2000s, before becoming more stable into the 2010s, from a multinational perspective. This observation is in line with those reported in previous reviews, which looked at trends in child and adolescent mental health problems between the 1950s and 2000s [3, 4].

Analysing the time trends in different multinational subregions showed that there are some small differences between the regions over time. Northern Europe was the only region that showed a clearly significant minor increasing trend, while not being the region with the highest total prevalence of PSHC at present. Based on the results of this systematic review and meta-analysis, and accounting for the all the increasing/decreasing trends between all nations within the included data, an overall significant minor increase in the prevalence of PSHC among adolescents can be observed between 1982 and 2013. This is likely to influence adolescent health, functioning and well-being, while the growing evidence of a trend of increasing burdens in the context of adolescent mental health is rightfully a public health concern, as stated by earlier research.

## Supporting information

S1 PRISMA checklist (DOC)

S1 Appendix Peer review of electronic search strategies (PRESS). (DOCX)

S2 Appendix Complete search strategy 22.11.2016. (DOCX)

S3 Appendix Risk of bias assessment. (DOCX)
