# Supplementary material for: Temporal trends in adolescents’ self-reported psychosomatic health complaints from 1980-2016: A systematic review and meta-analysis
Source: PLoS One. 2017 Nov 28;12(11):e0188374. doi: 10.1371/journal.pone.0188374 (PMC5705135; doi:10.1371/journal.pone.0188374)
Supplement: S1 Appendix — (DOCX) [file pone.0188374.s002.docx]

| **Peer Review of Electronic Search Strategies (PRESS)** | | | | | |
| --- | --- | --- | --- | --- | --- |
| **Review title:** Time trends in adolescent subjective health complaints from 1985-2015 | | | | | |
| **Author:** **Thomas Potrebny <Thomas.Potrebny@hib.no>** | | | **Reviewer:Regina Küfner Lein** | | **Date completed: 11. Nov. 2016** |
| **Database: Medline (Ovid)** | | | | | |
|  |  |  | | **If “B” or “C,” please provide an explanation or example:** | |
| **1** | **Translation of the research question** | A. No revisions  B. Revision(s) suggested  C. Revision(s) required | |  | |
| **2** | **Boolean and proximity operators** | A. No revisions  B. Revision(s) suggested  C. Revision(s) required | |  | |
| **3** | **Subject headings** | A. No revisions  B. Revision(s) suggested  C. Revision(s) required | |  | |
| **4** | **Text word searching** | A. No revisions  B. Revision(s) suggested  C. Revision(s) required | |  | |
| **5** | **Spelling, syntax, and line numbers** | A. No revisions  B. Revision(s) suggested  C. Revision(s) required | |  | |
| **6** | **Limits and filters** | A. No revisions  B. Revision(s) suggested  C. Revision(s) required | |  | |
| **7** | **Overall evaluation**  (if one or more «revision required is noted above, the response must be «revisions required») | A. No revisions  B. Revision(s) suggested  C. Revision(s) required | |  | |

**Additional comments:**

|  |
| --- |
