# Supplementary material for: Temporal trends in adolescents’ self-reported psychosomatic health complaints from 1980-2016: A systematic review and meta-analysis
Source: PLoS One. 2017 Nov 28;12(11):e0188374. doi: 10.1371/journal.pone.0188374 (PMC5705135; doi:10.1371/journal.pone.0188374)
Supplement: S2 Appendix — (DOCX) [file pone.0188374.s003.docx]

**Appendix 2. Complete search strategy 22.11.2016.**

Database: Epub Ahead of Print, In-Process & Other Non-Indexed Citations, Ovid MEDLINE(R) Daily and Ovid MEDLINE(R) <1946 to Present>

Search Strategy:

--------------------------------------------------------------------------------

1 Diagnostic Self Evaluation/ or Psychophysiologic Disorders/ or Psychosomatic Medicine/ (25966)

2 ((subjective or self-reported) adj3 (health or complaints)).ti,ab. (12319)

3 (health complaint* or psychosomatic or psychophysiolog*).ti,ab. (22171)

4 adolescent/ or child/ or Young Adult/ (2910380)

5 (adolescen* or youth or youths or kid or kids or preteen or teen* or child* or young or juvenile).ti,ab. (1757265)

6 time/ or time factors/ (1169764)

7 (time or trend or trends or secular or temporal).ti,ab. (3087898)

8 1 or 2 or 3 (51720)

9 4 or 5 (3659872)

10 6 or 7 (3919093)

11 8 and 9 and 10 (2350)

***************************

Database: Embase <1974 to 2016 Week 47>

Search Strategy:

--------------------------------------------------------------------------------

1 self evaluation/ (27812)

2 (Diagnostic Self Evaluation or self diagnosis).ti,ab. (397)

3 psychophysiology/ (19571)

4 psychosomatic disorder/ or psychosomatics/ (29139)

5 (health complaint* or psychosomatic or psychophysiolog*).ti,ab. (28977)

6 ((subjective or self-reported) adj3 (health or complaints)).ti,ab. (13682)

7 adolescent/ (1401923)

8 child/ (1630709)

9 juvenile/ (60856)

10 young adult/ (150665)

11 (adolescen* or youth or youths or kid or kids or preteen or teen* or child* or young or juvenile).ti,ab. (1999315)

12 time/ or time factor/ (393696)

13 trend study/ (18273)

14 (time or trend or trends or secular or temporal).ti,ab. (3683026)

15 1 or 2 or 3 or 4 or 5 or 6 (101951)

16 7 or 8 or 9 or 10 or 11 (3243179)

17 12 or 13 or 14 (3954500)

18 15 and 16 and 17 (3341)

***************************

Database: PsycINFO <1967 to November Week 2 2016>

Search Strategy:

--------------------------------------------------------------------------------

1 Health Complaints/ or Self-Report/ or exp Psychophysiology/ (23075)

2 psychosomatic medicine/ (1556)

3 ((subjective or self-reported) adj3 (health or complaints)).ti,ab. (5507)

4 (health complaint* or psychosomatic or psychophysiolog*).ti,ab. (20457)

5 exp Somatoform Disorders/ (11229)

6 ADOLESCENT PSYCHOLOGY/ or Adolescent Characteristics/ (3938)

7 child characteristics/ or child psychology/ (4195)

8 (adolescen* or youth or youths or kid or kids or preteen or teen* or child* or young or juvenile).ti,ab. (773874)

9 TIME/ (11511)

10 TRENDS/ (9398)

11 (time or trend or trends or secular or temporal).ti,ab. (572556)

12 1 or 2 or 3 or 4 or 5 (51394)

13 6 or 7 or 8 (774171)

14 9 or 10 or 11 (575546)

15 12 and 13 and 14 (1261)

16 12 and 14 (7125)

17 limit 16 to (180 school age <age 6 to 12 yrs> or 200 adolescence <age 13 to 17 yrs> or 320 young adulthood <age 18 to 29 yrs>) (2186)

18 15 or 17 (2647)

***************************
