# Supplementary material for: Temporal trends in adolescents’ self-reported psychosomatic health complaints from 1980-2016: A systematic review and meta-analysis
Source: PLoS One. 2017 Nov 28;12(11):e0188374. doi: 10.1371/journal.pone.0188374 (PMC5705135; doi:10.1371/journal.pone.0188374)
Supplement: S3 Appendix — (DOCX) [file pone.0188374.s004.docx]

**Appendix 3. Risk of bias assessment.**

| Criteria | Was the sample frame appropriate to address the target population? | Were study participants sampled in an appropriate way? | Was the sample size adequate? | Were the study subjects and the setting described in detail? | Was the data analysis conducted with sufficient coverage of the identified sample? | Were valid methods used for the identification of the condition? | Was the condition measured in a standard, reliable way for all participants? | Was there appropriate statistical analysis? | Was the response rate adequate, and if not, was the low response rate managed appropriately? |
| --- | --- | --- | --- | --- | --- | --- | --- | --- | --- |
| Achenbach et al. (2002) | Y | Y | Y | Y | Y | Y | Y | U | Y |
| Berntsson et al. (2001) | Y | Y | Y | Y | Y | Y | Y | Y | Y |
| Berntsson et al. (2014) | Y | Y | Y | Y | Y | Y | Y | Y | N*(Y) |
| Braverman et al. (2016) | Y | Y | Y | Y | Y | Y | Y | Y | Y |
| Dey et al. (2015) | Y | Y | Y | Y | Y | Y | Y | Y | Y |
| Due et al. (2003) | Y | Y | Y | Y | Y | Y | Y | N | Y |
| Duinhof et al. (2014) | Y | Y | Y | Y | Y | Y | Y | Y | Y |
| Fink et al. (2015) | N | U | Y | Y | N | Y | Y | Y | Y |
| Fleming et al. (2014) | N | Y | Y | Y | U | Y | Y | Y | Y |
| Hagquist (2009) | Y | Y | Y | Y | Y | Y | Y | Y | Y |
| Henriksen et al. (2012) | N | U | U | N | N | Y | U | Y | Y |
| Levin et al. (2009) | Y | Y | Y | U | Y | Y | Y | Y | Y |
| Levin et al. (2015) | Y | Y | Y | Y | Y | Y | Y | Y | Y |
| Maughan et al. (2012) | Y | Y | Y | Y | Y | Y | Y | Y | Y |
| Norell-Clarke et al. (2016) | Y | Y | Y | Y | Y | Y | Y | Y | Y |
| Ottová-Jordan et al. (2015a) | Y | Y | Y | U | Y | Y | Y | Y | U |
| Ottová-Jordan et al. (2015b) | Y | Y | Y | U | Y | Y | Y | Y | U |
| Sourander et al. (2012) | Y | Y | Y | Y | U | Y | Y | Y | Y |
| Tick et al. (2008) | N | Y | Y | Y | N | Y | Y | Y | Y |
| Twenge (2015) | Y | Y | Y | Y | Y | U | Y | N | Y |
| van Geelen et al. (2016) | Y | Y | Y | Y | Y | Y | Y | Y | Y |

Y = Yes; N = No; U = Unclear; NA = Not applicable

**Comments**

There were several relevant notes on the quality of some studies regarding design, analysis and sampling. Regarding response rate, Berntsson and Ringberg [34] had a response rate of 45.7 % (n = 1461) but conducted a nonresponse analysis and weights were calibrated, allowing the sample to be representative at a national level, according to the authors themselves. There were also two instances of unknown response rate for Ottova-Jordan and colleagues [8, 24], following the protocol of the HBSC however, these are likely to be adequate.

The JBI manual [26] states that reporting of the statistical analysis should include the numerator and denominator and that percentages should be given with confidence intervals. Furthermore, the methods section should be detailed enough for reviewers to identify the analytical technique used and how specific variables were measured. Three studies did not clearly report details from the performed statistical analysis. Due and colleagues [36] did not provide confidence intervals (or other measure of variance or effect size) for the proportions of their measure of PSHC. Twenge [48] and Achenbach and colleagues [33] did not present the statistical analysis for PSHC in sufficient detail (does not provide enough details about what is presented in result and tables).

Regarding sampling, Fleming and colleagues [39] note that there were some variations between their samples of 2007 and 2012. It is unsure if the changes are caused by different sampling or changes in the demography of secondary school. Tick and colleagues [47] had some limitations regarding nonresponding non-Dutch speakers, which limits the generalizability of results to the Dutch speaking part of the population. For Fink and colleagues [38], a key limitation of the current sample was that it was not selected at random and so may not be fully representative of the wider adolescent community. Furthermore, the ethnicity of students in this sample is over representative of adolescents from minority ethnic groups.

Henriksen and colleagues [41] had low overall study quality. There are several notes on the study quality, first the details of recruitment of the school from 2010 are unclear and therefore it is unclear if the study participants were recruited in an appropriate way. Moreover, it is not clear if there was a sample size calculation to determine an adequate sample size. Second, there were differences between the two schools that were studied: one school was rural and the other was urban, and the response rates at each of the two time-points (1996 and 2010) differed significantly. Third, the measurement scale was updated and several items were changed for the 2010 sample, making comparability questionable. It is therefore unclear if this might have had an influence on the results or not, even though the intention was to improve the scale overall.
